# Comparability of six different immunoassays measuring SARS‐CoV‐2 antibodies with neutralizing antibody levels in convalescent plasma: From utility to prediction

**DOI:** 10.1111/trf.16600

**Published:** 2021-08-17

**Authors:** Abigail Lamikanra, Dung Nguyen, Peter Simmonds, Sarah Williams, Emma M. Bentley, Cathy Rowe, Ashley David Otter, Tim Brooks, Kimberly Gilmour, Annabelle Mai, Jusvinder Dadhra, Mabel Csatari, Sheba Ziyenge, Marta Oliveira, Rutger Ploeg, Pat Tsang, Maria Zambon, Robin Gopal, Julie Huiyuan Xiao, Alain Townsend, David Roberts, Heli Harvala

**Affiliations:** ^1^ Clinical, Research and Development Departments NHS Blood and Transplant Oxford UK; ^2^ Nuffield Department of Medicine, Peter Medawar Building for Pathogen Research University of Oxford Oxford UK; ^3^ National Institute for Biological Standards and Control (NIBSC) South Mimms UK; ^4^ Rare and Imported Pathogens Laboratory Public Health England Porton Down UK; ^5^ Laboratory Services and NIHR BRC Departments Great Ormond Street Hospital London UK; ^6^ Nuffield Department of Surgical Sciences and BRC Surgical Theme University of Oxford Oxford UK; ^7^ Research Laboratory Department NHS Blood and Transplant Oxford UK; ^8^ Virology Reference Department National Infection Service, Public Health England London UK; ^9^ High Containment Microbiology Department, National Infection Service Public Health England London UK; ^10^ MRC Human Immunology Unit, MRC Weatherall Institute, Radcliffe Department of Medicine John Radcliffe Hospital Oxford UK; ^11^ Chinese Academy of Medical Science Oxford Institute, Nuffield Department of Medicine University of Oxford Oxford UK; ^12^ Radcliffe Department of Medicine and BRC Haematology Theme University of Oxford Oxford UK; ^13^ Microbiology Services NHS Blood and Transplant London UK

**Keywords:** infectious disease testing, intravenous immunoglobulin

## Abstract

**Background:**

Convalescent plasma (CP) therapy for coronavirus disease (COVID‐19) provides virus‐neutralizing antibodies that may ameliorate the outcome of severe acute respiratory syndrome coronavirus 2 (SARS‐CoV‐2) infections. The effectiveness of CP likely depends on its antiviral neutralizing potency and is determined using in vitro neutralizing antibody assays.

**Study design and methods:**

We evaluated abilities of three immunoassays for anti‐spike antibodies (EUROimmun, Ortho, Roche), a pseudotype‐based neutralization assay, and two assays that quantify ACE2 binding of spike protein (GenScript and hemagglutination test [HAT]‐based assay) to predict neutralizing antibody titers in 113 CP donations. Assay outputs were analyzed through linear regression and calculation of sensitivities and specificities by receiver operator characteristic (ROC) analysis.

**Results:**

Median values of plasma samples containing neutralizing antibodies produced conversion factors for assay unitage of ×6.5 (pseudotype), ×19 (GenScript), ×3.4 (HAT assay), ×0.08 (EUROimmun), ×1.64 (Roche), and ×0.10 (Ortho). All selected assays were sufficient in identifying the high titer donations based on ROC analysis; area over curve ranged from 91.7% for HAT and GenScript assay to 95.6% for pseudotype assay. However, their ability to predict the actual neutralizing antibody levels varied substantially as shown by linear regression correlation values (from 0.27 for Ortho to 0.61 for pseudotype assay).

**Discussion:**

Overall, the study data demonstrate that all selected assays were effective in identifying donations with high neutralizing antibody levels and are potentially suitable as surrogate assays for donation selection for CP therapy.

**Abbreviation**
SARS‐CoV‐2: COVID‐19; neutralising antibody testing; convalescent plasma

## 1 INTRODUCTION

During the first year of the COVID‐19 pandemic, there have been many challenges and efforts to control the spread of this disease and treat those infected. The emergence of new variants with greater transmission and increased potential for severe disease^1^, ^2^ indicates that protection and prompt treatment of immunocompromised individuals and poor vaccine responders remain necessary.

Convalescent plasma (CP) may supplement humoral immunity that is absent in populations most vulnerable to severe disease.^3^, ^4^ Effectiveness of therapeutic antibodies and CP to treat or prevent serious disease is dependent on the concentration of functional antibodies and their antigenic specificity and isotype.^5^, ^6^ CP can prevent severe disease if it contains sufficiently high antibody titers and is given within 4 days of displaying disease symptoms.^7^ However, antibody titers from patients who have recovered from coronavirus disease (COVID‐19) are highly variable,^6^, ^8^, ^9^ and the absence of internationally agreed methods to determine effective neutralizing titers of severe acute respiratory syndrome coronavirus‐2 (SARS‐CoV‐2) antibody prevents meaningful comparison of trial results from different study sites. This has hindered progress in drawing up guidelines on best practice and use of CP for COVID‐19.

There are increasingly supporting evidence to suggest that neutralizing antibody titers correlate with protection against COVID‐19.^10^, ^11^ Clinical trials that ended in futility may have used convalescent plasma with insufficient neutralizing antibody, for example, below the commonly used threshold titer of ≥1:100 as determined by live virus neutralization assays.^12^ However, such assays require high containment facilities and require greater time investment to establish than standard immunoassays. Here, we investigate the abilities of pseudotype, ACE‐2 blocking, and automated serological methods to predict neutralization titers and identify high titer units in a panel of convalescent serum samples with a range of anti‐SARS‐CoV‐2 reactivity.

## 2 MATERIALS AND METHODS

Convalescent plasma was collected from individuals with previous suspected or laboratory confirmed SARS‐CoV‐2 infections at least 28 days after the resolution of their symptoms between May 6, 2020 and May 12, 2020 (Table S1). Signed consent was obtained from each donor at the time of donation for holding information about them, their health, attendances, and donations. It also included the use of that data for the purpose of clinical audit to assess and improve the service provided by NHS Blood and Transplant as well as the use for research to improve our knowledge of the donor population.

SARS‐CoV‐2 neutralizing antibody titers in ethylenediamine tetraacetic acid (EDTA) blood samples collected from convalescent plasma donors were determined using a live virus microneutralization assay with the England‐2 SARS‐CoV‐2 strain as previously reported.^13^ Samples were tested in pseudotype neutralization assays using a construct that incorporated the SARS‐CoV‐2 spike protein expressed from a codon optimized Wuhan‐Hu‐1 synthetic gene sequence with the D614G substitution. EUROimmun IgG spike enzyme‐linked immunosorbent assay (ELISA; Lübeck, Germany), Vitros IgG spike assay (Ortho Clinical Diagnostics, Raritan, New Jersey), Elecsys anti‐SARS‐CoV‐2 S assay (Roche, Basel, Switzerland), and GenScript surrogate virus neutralization test (Piscataway, New Jersey) were performed as specified by the manufacturer. A hemagglutination test (HAT) was based on red cell agglutination to detect antibodies to receptor binding domain (RBD) of the SARS‐CoV‐2.^14^

All statistical analyses were performed using SPSS version 26.

## 3 RESULTS

### 3.1 Comparison of antibody quantitation in different assay formats

A total of 113 convalescent plasma samples were evaluated for reactivity in a SARS‐CoV‐2 neutralization assay using the England‐2 virus isolate. Forty‐four from 51 of the neutralization antibody‐positive samples had serologically confirmed or probable previous SARS‐CoV‐2 infections (86%) compared with 36 of 62 (58%) without confirmation (Table S1). Neutralizing antibody titers, representing the interpolated titer causing a 50% reduction (IC_50_) in virus replication ranged from undetectable (<1:16) to 1:1600. Reactivity was compared with titers similarly based on interpolated IC_50_ values in a pseudotype neutralization assay. Further comparisons were made with two assays measuring inhibition of spike protein binding to ACE2 (GenScript, HAT) and three commercially available direct binding ELISAs based on recombinant antigens derived from human cells (EUROimmun, Roche, Ortho).

**FIGURE 1:**
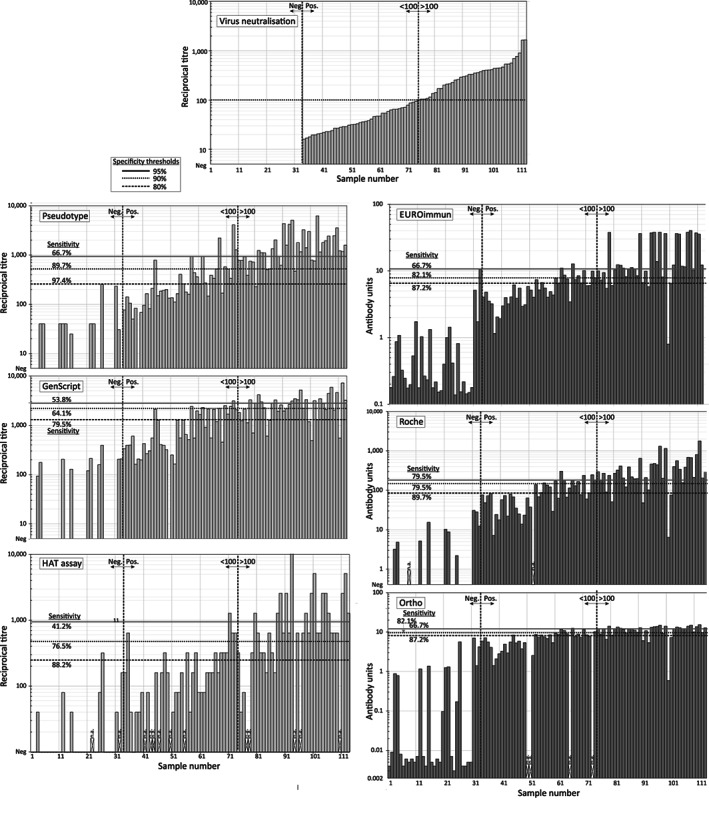
Reactivities of the plasma panel in neutralization, ACE‐2 blocking assay, and immunoassays. Reactivities of the test samples in serological assays. Samples (1–113) were ranked by NAb titer (top center) and plotted in the same order in other serological tests (left, pale blue: neutralization or ACE2‐blocking assays, right, pale orange: immunoassay for IgG to spike protein). The negative, positive, and 1:100 titer thresholds in the neutralizing antibody (Nab) assay were indicated by dotted blue vertical lines. The 1:100 threshold was used in ROC analysis (Figure 3) to determine assay sensitivity at selected specificity values relative to the NAb assay. The frequency of samples with reactivities above each specificity value to the right of the 1:100 threshold line depicts sensitivity (as reported in the ROC analysis); specificity indicated to the left. Red cross: Sample not available for testing. ROC, receiver operator characteristic

To compare reactivities, samples were ranked by neutralizing antibody titer and output values from other tests (IC_50_ in pseudotype and GenScript assay, inhibition titers in the HAT assay, signal to cutoff ratios in the spike protein immunoassays) plotted in the same order (Figure 1). Antibody detection in each assay showed a degree of comparability with live virus neutralizing antibody testing, with largely absent or low reactivity of samples that were negative with microneutralization test and evident trends for increasing assay values in positive samples; nonparametric rank‐order testing demonstrated significant associations between virus neutralization titers and each test (Table S2).

Antibody titers in virus neutralization, pseudotype, and HAT assays were approximately normally distributed (Table S3), although antibody quantification in ELISAs typically deviated from normality because of compression of values from high titer samples. Antibody levels measured in each assay showed relatively high correlation coefficients on linear regression (Table 1), comparable to those of other models with greater parameterization (e.g., logarithmic, inverse, quadratic and cubic, Table S4). The following analyses assume linearity although we acknowledge this can only be an approximation given the compression of values from the ELISAs.

**TABLE 1:** Antibody quantitation and NAb titer prediction by each serology assay

Assay	Factor^c^	Conversion	Correlation (*R* ^2^)^a^	NAb titer >1/100^b^	Sensitivity
		All	Pos	Threshold	
NAb	(×1.0)	(1.0)	(1.0)	(1:100)	(100%)
Pseudotype	×6.5	0.78	0.61	1:518	89.7%
GenScript	×19	0.67	0.40	2127	64.1%
HAT assay	×3.4	0.72	0.54	1:480	76.5%
Roche S	×1.64	0.71	0.43	169.5	79.5%
EuroImmun	×0.08	0.68	0.42	7.98	82.1%
Ortho	×0.10	0.53	0.27	9.15	82.1%

^a^ Pearson correlation coefficients between test assay quantitation and NAb titers using parametric linear regression of log‐transformed values for all samples (All) and for the subset of samples shown to contain neutralizing antibodies (Pos).

^b^ Thresold values and proportion of samples detected (sensitivity) at 90% specificity.

^c^ Ratio of median test value of positive samples to NAb titers.

**FIGURE 2:**
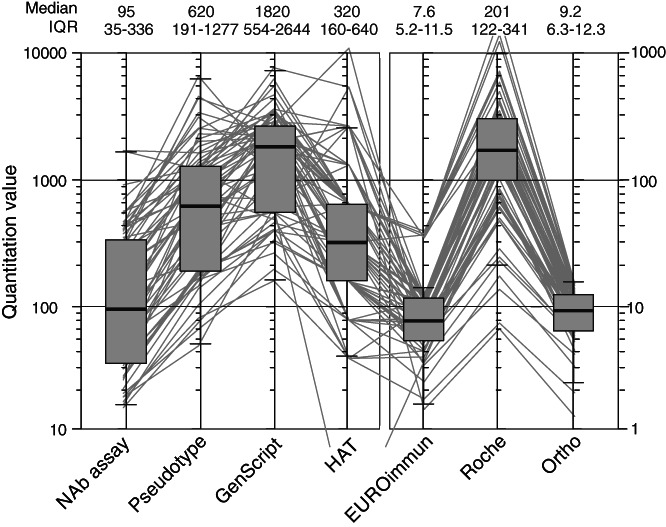
Comparison of assay values (median and ranges) of SARS‐CoV‐2 serology assays. Comparison of reactivities of the NAb‐positive test samples in each of the serology assay expressed in their own assay units, with a Tukey's plot superimposed to show median and range of assay values. Medians, IQRs, and correlation coefficients between each assay with NAb titers are shown above the graph. The *y*‐axis on the right is for EUROimmun, Roche, and Ortho values. IQR, interquartile range

To quantify the interconvertibility of outputs produced by the different serological assays, the median values and interquartile ranges of the 81 samples with detectable neutralizing antibodies were compared (Figure 2; Table 1). A median neutralizing antibody titer of 1:95 equated with 1:620 in the pseudotype assay indicating a conversion factor of ×6.5 for comparing results from the two assays (Table 1, column 2). Titers in GenScript and HAT assay showed similar differences in median values and associated conversion factors. An extended comparison of titers and reactivities between each assay pair showed that quantitation was dependent in part on assay formats (Table S5). For example, the values obtained from the three immunoassays (Roche, Ortho, and EUROimmun) correlated closely with each other (all *R* ^2^ values >0.86), but less well with titers in the neutralizing antibody testing and pseudotype assays (*R* ^2^ values <0.65). Anti‐spike blocking detected in the HAT assay similarly correlated poorly with immunoassay results (*R* ^2^ values <0.38) but better with live virus and pseudotype neutralization titers (0.73 and 0.67, respectively), although its correlation with quantitation by the GenScript assay that also quantifies blocking of spike/ACE receptor binding was lower (0.46).

### 3.2 Evaluation of each assay's ability to detect high neutralizing antibody titer samples

Pseudotype, ACE2 blocking assays, and immunoassays based on the spike gene have been widely used as surrogate assays to identify samples with high neutralizing antibody titers for use in immunotherapy. The effect of threshold specificity values on detection rates can be visualized as the proportion of correctly and incorrectly identified samples in the ranking analysis (Figure 1; specificity thresholds of 80%, 90%, and 95% are shown). With this information, a threshold of 90% specificity, serology assays showed between 64% (GenScript) and 90% (pseudotype) detection frequencies of the samples with >1:100 titers (Table 1).

**FIGURE 3:**
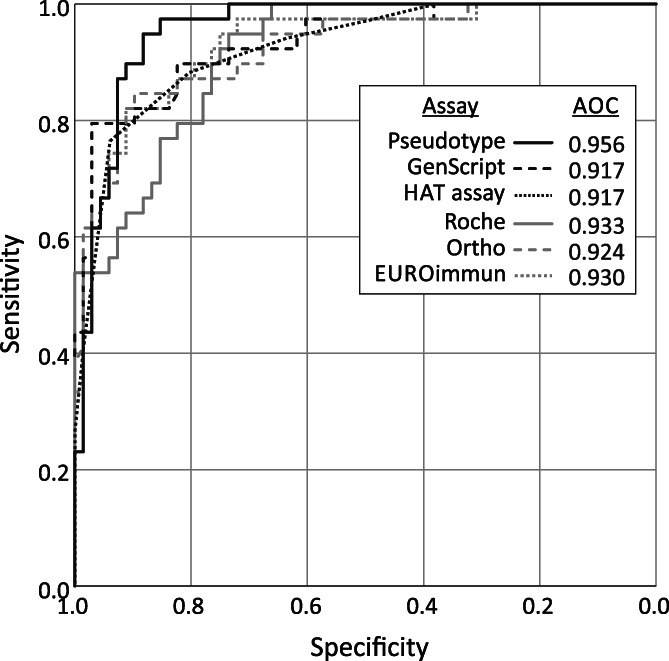
Sensitivity and specificity of serological assays to predict titers >1:100 in neutralizing antibody assay. ROC analysis of reactivities of NAb‐assay‐positive samples in other serological tests. Graph lines for each test represent specificities (*x*‐axis; reverse scale) and sensitivities (*y*‐axis) for detection of samples with >1/100 NAb titers. AOC, Area over curve; higher values denote greater predictive ability. ROC, receiver operator characteristic

To compare their predictive abilities, reactivities of the 81 samples with detectable neutralizing antibodies in each assay were compared by receiver operator characteristic (ROC) analysis (Figure 3). Each assay was analyzed for its ability to detect the subset of 40 samples with neutralizing antibody levels higher than 1:100, expressed in terms of specificity and sensitivity. All assays could display 100% sensitivity (ability to detect all samples with titers >1:100) and 100% specificity (avoidance of samples with <1:100 titers), but these requirements were conflicting with markedly different reactivity values required to meet these thresholds. The area over the curve (AOC) provides a combined metric of specificity and sensitivity, with the pseudotype assay coming closest to reproducing the results from the NAb assay (AOC = 0.96).

## 4 DISCUSSION

We compared antibody quantitation by pseudotype, ACE‐2 blocking, or spike binding antibody assays to evaluate their suitability as surrogate assays for measuring virus neutralization levels and for reliable identification of convalescent plasma unit with high titers. We recognize that the ELISAs analyzed in the study are primarily designed to provide qualitative results and that there are inherent limitations in this format associated with signal saturation for samples with high antibody levels. Nevertheless, we demonstrated that all assays reliably identified donations with high neutralizing antibody levels (>1:100, Table 1), with assay cutoffs of >8.0 S/Co (EUROimmun), >170 U/ml (Roche), S/Co >9.1 (Ortho), and titers >518 (>66% inhibition; GenScript) predictive of high titer (>1:100) neutralizing antibodies in convalescent plasma with 90% specificity.

The US Food and Drug Administration (FDA) issued previously a guidance for the emergency use of convalescent plasma for treatment of hospitalized patients with COVID‐19.^15^ This proposed cutoffs for 9 commercial serological assays for identification of high titer units. Cutoffs of 9.5 for Ortho assay and 68% inhibition for GenScript assay were comparable to those we obtained, but their proposed S/Co of >3.5 in the EUROimmun assay conflicts with the results of ROC analysis in the current study. Samples with reactivity above the threshold would have a predicted specificity of 46% for neutralizing antibody titers >1:100 (Table S1), substantially lower than the >90% specificity using an S/Co ratio of 8.0 we determined. The proposed S/Co ratio of 132 U/ml for Roche Elecsys assay in the FDA study would similarly lead to reduced specificity (84%).

Although live virus neutralization assays are regarded as the gold standard assay for determining neutralizing antibody titers, it requires highly trained personnel to work at biosafety level 3; assay quantitation between laboratories has also not been standardized to date. Virus neutralizing assays would be expensive and time consuming to implement for screening of thousands of samples often required for clinical studies.^16^, ^17^ For these reasons, we have tried to assess which of the currently available assays would best predict the neutralizing antibody levels in high titer donations and might be used as surrogate tests. Although we have not assessed intra‐ and inter‐assay variability in this study, pseudotype and HAT assays showed the best correlations with neutralizing antibody levels obtained with live virus assay; the good correlation of results from the GenScript assay (*R* ^2^ = 0.67) was consistent in comparison with GenScript titers with those achieving 50% and 90% plaque reduction in a microneutralization assay (*R* ^2^ = 0.53 and *R* ^2^ = 0.46, respectively).^18^ Of these, the HAT assay is simpler to run and can also be easily introduced also in low‐income countries as it does not require any complicated or expensive laboratory machinery. Although existing qualitative ELISAs were effective for identifying samples with high neutralizing antibody titers, the development of new ELISAs specifically designed to quantity antibody levels over a wider dynamic range may offer substantially enhanced predictive ability and their potential wider use for evaluation of humoral immunity in those infected or immunized with SARS‐CoV‐2.

Our study highlights the need for assay cross‐comparisons and robust metrics of correlations with the virus neutralization assay to enable their use as surrogate assays for detection and quantification of high titer samples. Assay comparisons will be of further value in the standardization of assay outputs and comparison of anti‐SARS‐CoV‐2 antibody data generated worldwide.

## CONFLICT OF INTEREST

The authors have disclosed no conflicts of interest.

## Supporting information


**Table S1.** Donor demographics for the study group.
**Table S2**. Assocations between virus neutralising antibody titres and antibody quantitation in other assays by spearman rank correlation test.
**Table S3**. Normality testing for log transformed antibody quantitation in the serological assays.
**Table S4**. Correlations of assay values with neutralising antibody titres using different regression models.
**Table S5**. Inter‐assay correlations of reactivity of samples containing neutralising antibodies.
