# Supplementary material for: Comparability of six different immunoassays measuring SARS‐CoV‐2 antibodies with neutralizing antibody levels in convalescent plasma: From utility to prediction
Source: Transfusion. 2021 Aug 17;61(10):2837–43. doi: 10.1111/trf.16600 (PMC8447482; doi:10.1111/trf.16600)
Supplement: Supplementary file 1 — Table S1. Donor demographics for the study group. Table S2. Assocations between virus neutralising antibody titres and antibody quantitation in other assays by spearman rank correlation test. Table S3. Normality testing for log transformed antibody quantitation in the serological assays. Table S4. Correlations of assay values with neutralising antibody titres using different regression models. Table S5. Inter‐assay correlations of reactivity of samples containing neutralising antibodies. [file TRF-61-2837-s001.docx]

**SUPPLEMENTARY DATA**

**TABLE S1**

DONOR DEMOGRAPHICS FOR THE STUDY GROUP

| **Study_No** | **Sample _date**  **(mm/yyyy)** | **Sero_conf^1^** | **Days from dg^2^** | **Sex** | **Virus_Neut** |
| --- | --- | --- | --- | --- | --- |
| 1 | 06/2020 | ND | ND | M | <16 |
| 2 | 06/2020 | ND | ND | M | <16 |
| 3 | 06/2020 | Certain | 36 | F | <16 |
| 4 | 07/2020 | Certain | 43 | F | <16 |
| 5 | 07/2020 | ND | ND | M | <16 |
| 6 | 07/2020 | ND | ND | M | <16 |
| 7 | 07/2020 | ND | ND | M | <16 |
| 8 | 07/2020 | ND | ND | M | <16 |
| 9 | 07/2020 | ND | ND | M | <16 |
| 10 | 07/2020 | ND | ND | M | <16 |
| 11 | 08/2020 | ND | ND | M | <16 |
| 12 | 08/2020 | ND | ND | M | <16 |
| 13 | 08/2020 | ND | ND | M | <16 |
| 14 | 08/2020 | ND | ND | M | <16 |
| 15 | 08/2020 | ND | ND | M | <16 |
| 16 | 08/2020 | ND | ND | M | <16 |
| 17 | 08/2020 | ND | ND | M | <16 |
| 18 | 08/2020 | ND | ND | M | <16 |
| 19 | 09/2020 | ND | ND | M | <16 |
| 20 | 10/2020 | ND | ND | M | <16 |
| 21 | 10/2020 | Certain | 39 | F | <16 |
| 22 | 11/2020 | Certain | 34 | M | <16 |
| 23 | 11/2020 | Probable | ND | M | <16 |
| 24 | 11/2020 | ND | ND | M | <16 |
| 25 | 11/2020 | ND | ND | M | <16 |
| 26 | 12/2020 | ND | ND | M | <16 |
| 27 | 12/2020 | ND | ND | M | <16 |
| 28 | 12/2020 | ND | ND | M | <16 |
| 29 | 12/2020 | ND | ND | M | <16 |
| 30 | 11/2020 | ND | ND | M | <16 |
| 31 | 12/2020 | Probable | ND | F | <16 |
| 32 | 12/2020 | Certain | 58 | M | <16 |
| 33 | 12/2020 | ND | ND | M | <16 |
| 34 | 11/2020 | Certain | 66 | M | 16 |
| 35 | 07/2020 | ND | ND | M | 17 |
| 36 | 06/2020 | ND | ND | M | 18 |
| 37 | 11/2020 | Certain | 63 | F | 20 |
| 38 | 12/2020 | ND | ND | M | 20 |
| 39 | 11/2020 | ND | ND | M | 21 |
| 40 | 05/2020 | ND | ND | F | 21 |
| 41 | 12/2020 | Certain | 63 | M | 22 |
| 42 | 11/2020 | Certain | 41 | M | 23 |
| 43 | 12/2020 | Certain | 52 | M | 23 |
| 44 | 12/2020 | Certain | 40 | M | 24 |
| 45 | 08/2020 | ND | ND | M | 27 |
| 46 | 05/2020 | ND | ND | M | 27 |
| 47 | 07/2020 | Certain | 39 | M | 28 |
| 48 | 12/2020 | ND | ND | M | 29 |
| 49 | 05/2020 | ND | ND | M | 29 |
| 50 | 12/2020 | Certain | 43 | F | 31 |
| 51 | 11/2020 | ND | ND | M | 32 |
| 52 | 11/2020 | ND | ND | M | 32 |
| 53 | 08/2020 | Probable | ND | M | 33 |
| 54 | 06/2020 | ND | ND | M | 35 |
| 55 | 11/2020 | Certain | 48 | M | 36 |
| 56 | 07/2020 | Certain | 46 | F | 37 |
| 57 | 11/2020 | Probable | ND | M | 38 |
| 58 | 07/2020 | Certain | 42 | M | 41 |
| 59 | 06/2020 | Probable | ND | F | 46 |
| 60 | 11/2020 | ND | ND | M | 47 |
| 61 | 11/2020 | Certain | 49 | F | 47 |
| 62 | 09/2020 | Certain | 46 | M | 54 |
| 63 | 05/2020 | Certain | 41 | F | 54 |
| 64 | 11/2020 | ND | ND | M | 59 |
| 65 | 06/2020 | Certain | 40 | M | 63 |
| 66 | 07/2020 | Probable | ND | M | 65 |
| 67 | 11/2020 | ND | ND | M | 65 |
| 68 | 12/2020 | Certain | 51 | F | 67 |
| 69 | 12/2020 | ND | ND | M | 70 |
| 70 | 11/2020 | ND | ND | M | 71 |
| 71 | 07/2020 | ND | ND | M | 79 |
| 72 | 07/2020 | ND | ND | M | 87 |
| 73 | 09/2020 | ND | ND | M | 91 |
| 74 | 06/2020 | Certain | 48 | F | 95 |
| 75 | 11/2020 | ND | ND | F | 102 |
| 76 | 07/2020 | Probable | ND | M | 104 |
| 77 | 08/2020 | Certain | 55 | F | 104 |
| 78 | 11/2020 | Certain | 39 | M | 108 |
| 79 | 12/2020 | ND | ND | M | 115 |
| 80 | 06/2020 | Certain | 37 | M | 136 |
| 81 | 07/2020 | Certain | 48 | M | 142 |
| 82 | 07/2020 | Certain | 38 | F | 171 |
| 83 | 07/2020 | Certain | 37 | M | 172 |
| 84 | 10/2020 | Probable | ND | M | 200 |
| 85 | 11/2020 | ND | ND | M | 210 |
| 86 | 11/2020 | ND | ND | F | 214 |
| 87 | 08/2020 | ND | ND | M | 228 |
| 88 | 11/2020 | ND | ND | M | 254 |
| 89 | 11/2020 | Probable | ND | M | 260 |
| 90 | 11/2020 | ND | ND | M | 288 |
| 91 | 10/2020 | ND | ND | M | 300 |
| 92 | 10/2020 | ND | ND | M | 308 |
| 93 | 12/2020 | ND | ND | M | 333 |
| 94 | 07/2020 | Certain | 39 | M | 336 |
| 95 | 10/2020 | Certain | 46 | M | 353 |
| 96 | 08/2020 | Certain | 39 | M | 358 |
| 97 | 09/2020 | Certain | 46 | M | 376 |
| 98 | 11/2020 | ND | ND | M | 395 |
| 99 | 11/2020 | Probable | ND | M | 404 |
| 100 | 12/2020 | Certain | 59 | F | 409 |
| 101 | 06/2020 | Certain | 35 | M | 414 |
| 102 | 11/2020 | Certain | 42 | M | 440 |
| 103 | 11/2020 | Certain | 34 | M | 443 |
| 104 | 06/2020 | ND | ND | M | 448 |
| 105 | 06/2020 | ND | ND | M | 466 |
| 106 | 06/2020 | ND | ND | M | 536 |
| 107 | 10/2020 | Certain | 43 | M | 538 |
| 108 | 08/2020 | Certain | 38 | F | 561 |
| 109 | 11/2020 | ND | ND | M | 698 |
| 110 | 07/2020 | Certain | 48 | M | 772 |
| 111 | 06/2020 | ND | ND | M | 899 |
| 112 | 06/2020 | Certain | 40 | M | 1642 |
| 113 | 06/2020 | Certain | 36 | M | 1658 |

^1^Serologically confirmed SARS-CoV-2 infections; ND: not confirmed

^2^Days from confirmed SARS-CoV-2 infection

**TABLE S2**

ASSOCATIONS BETWEEN VIRUS NEUTRALISING ANTIBODY TITRES AND ANTIBODY QUANTITATION IN OTHER ASSAYS BY SPEARMAN RANK CORRELATION TEST

|  | **Neutralising antibody pos samples** | | | **All samples** | | |
| --- | --- | --- | --- | --- | --- | --- |
| **Assay** | **n** | **R^1^** | ***P^2^*** | **n** | **R^1^** | ***P^2^*** |
| PT | 81 | 0.833 | 5.83 x 10^-22^ | 114 | 0.915 | 5.59 x 10^-46^ |
| Genscript | 81 | 0.702 | 2.86 x 10^-13^ | 114 | 0.872 | 1.48 x 10^-36^ |
| HAT | 70 | 0.787 | 7.09 x 10^-16^ | 101 | 0.885 | 1.04 x 10^-34^ |
| EuroImmun | 81 | 0.757 | 2.78 x 10^-16^ | 114 | 0.867 | 1.34 x 10^-35^ |
| Roche | 79 | 0.719 | 8.79 x 10^-14^ | 111 | 0.88 | 5.32 x 10^-37^ |
| Ortho | 76 | 0.72 | 2.21 x 10^-13^ | 109 | 0.87 | 1.02 x 10^-34^ |

^1^Spearman rho

^2^Two tailed significance value

**TABLE S3**

NORMALITY TESTING FOR LOG TRANSFORMED ANTIBODY QUANTITATION IN THE SEROLOGICAL ASSAYS

|  | **Kolmogorov-Smirnov Test^1^** | | |
| --- | --- | --- | --- |
| **Assay** | **Test Statistic** | **DF^2^** | ***P^3^*** |
| Virus neut. | 0.09 | 65 | **0.2** |
| PT | 0.101 | 65 | **0.0947** |
| Genscript | 0.182 | 65 | <0.001 |
| HAT | 0.114 | 65 | 0.0354 |
| EuroImmun | 0.158 | 65 | 0.0003 |
| Roche | 0.087 | 65 | **0.2** |
| Ortho | 0.166 | 65 | 0.0001 |

^1^Lilliefors Significance Correction

^2^Degrees of freedom

^3^Normally distributed assay quantitation shown in bold

**TABLE S4**

CORRELATIONS OF ASSAY VALUES WITH NEUTRALISING ANTIBODY TITRES USING DIFFERENT REGRESSION MODELS

A) *All samples*

| **Curve** | **PT** | **Genscript** | **HAT** | **Roche** | **EuroImmun** | **Ortho** |
| --- | --- | --- | --- | --- | --- | --- |
| Linear | 0.783 | 0.673 | 0.724 | 0.713 | 0.684 | 0.537 |
| Logarithmic | 0.827 | 0.755 | 0.765 | 0.796 | 0.755 | 0.643 |
| Inverse | 0.807 | 0.776 | 0.755 | 0.82 | 0.765 | 0.701 |
| Quadratic | 0.834 | 0.776 | 0.764 | 0.812 | 0.767 | 0.699 |
| Cubic | 0.835 | 0.779 | 0.766 | 0.819 | 0.768 | 0.721 |
| Compound | 0.68 | 0.569 | 0.53 | 0.604 | 0.536 | 0.455 |
| Power | 0.761 | 0.665 | 0.628 | 0.712 | 0.632 | 0.556 |
| S | 0.78 | 0.709 | 0.678 | 0.765 | 0.679 | 0.617 |
| Growth | 0.68 | 0.569 | 0.53 | 0.604 | 0.536 | 0.455 |
| Exponential | 0.68 | 0.569 | 0.53 | 0.604 | 0.536 | 0.455 |
| Logistic | 0.68 | 0.569 | 0.53 | 0.604 | 0.536 | 0.455 |

B) *Neutralising antibody positive samples*

| **Curve** | **PT** | **Genscript** | **HAT** | **Roche** | **EuroImmun** | **Ortho** |
| --- | --- | --- | --- | --- | --- | --- |
| Linear | 0.607 | 0.4 | 0.538 | 0.427 | 0.42 | 0.273 |
| Logarithmic | 0.651 | 0.436 | 0.544 | 0.445 | 0.447 | 0.306 |
| Inverse | 0.674 | 0.458 | 0.53 | 0.45 | 0.458 | 0.333 |
| Quadratic | 0.684 | 0.468 | 0.547 | 0.452 | 0.467 | 0.346 |
| Cubic | 0.684 | 0.475 | 0.552 | 0.452 | 0.47 | 0.371 |
| Compound | 0.511 | 0.27 | 0.162 | 0.381 | 0.374 | 0.237 |
| Power | 0.558 | 0.295 | 0.179 | 0.403 | 0.405 | 0.268 |
| S | 0.59 | 0.311 | 0.192 | 0.413 | 0.423 | 0.293 |
| Growth | 0.511 | 0.27 | 0.162 | 0.381 | 0.374 | 0.237 |
| Exponential | 0.511 | 0.27 | 0.162 | 0.381 | 0.374 | 0.237 |
| Logistic | 0.511 | 0.27 | 0.162 | 0.381 | 0.374 | 0.237 |

**TABLE S5**

INTER-ASSAY CORRELATIONS OF REACTIVITY OF SAMPLES CONTAINING NEUTRALISING ANTIBODIES

| PT | R^1^ | 0.779 |  |  |  |  |  |
| --- | --- | --- | --- | --- | --- | --- | --- |
|  | *P^2^* | 1 x 10^-17^ |  |  |  |  |  |
| GenScript | R | 0.632 | 0.671 |  |  |  |  |
|  | *p* | 2.4 x 10^-10^ | 7.2 x 10^-12^ |  |  |  |  |
| HAT | R | 0.733 | 0.665 | 0.463 |  |  |  |
|  | *p* | 5.3 x 10^-13^ | 3.4 x 10^-10^ | 5.4 x 10^-5^ |  |  |  |
| EuroImmun | R | 0.648 | 0.581 | 0.621 | 0.375 |  |  |
|  | *p* | 6.0 x 10^-11^ | 1.3 x 10^-8^ | 5.0 x 10^-10^ | <10^-30^ |  |  |
| Roche | R | 0.654 | 0.564 | 0.623 | 0.263 | 0.898 |  |
|  | *p* | 6.5 x 10^-11^ | 6.1 x 10^-8^ | 8.5 x 10^-10^ | 0.03 | 3.0 x 10^-29^ |  |
| Ortho | R | 0.523 | 0.521 | 0.703 | 0.262 | 0.863 | 0.87 |
|  | *p* | 1.3 x 10^-6^ | 1.4 x 10^-6^ | 1.4 x 10^-12^ | 0.03 | 1..2 x 10^-23^ | 3.9 x 10^-24^ |
|  |  | NAb | PT | GenScript | HAT | EuroImmun | Roche |

^1^Pearson correlation coefficients; yellow: > 0.7; orange: >0.5; grey: < 0.5

^2^Two sided significance value
